# Endovascular neural stimulation with platinum and platinum black modified electrodes

**DOI:** 10.1038/s41598-025-93941-2

**Published:** 2025-03-20

**Authors:** Alexander R. Harris, Marko Ruslim, Huakun Xin, Zhiyi Shen, JingYang Liu, Tom Spencer, David Garrett, David B. Grayden, Sam E. John

**Affiliations:** 1 Department of Biomedical Engineering, University of Melbourne, Melbourne, 3010 Australia; 2 School of Engineering, RMIT University, Melbourne, VIC 3001 Australia; 3 Graeme Clark Institute, University of Melbourne, Melbourne, 3010 Australia

**Keywords:** Platinum black, Chronopotentiometric voltage transients, Charge transfer mechanism, Endovascular neural interface, Stent-electrode array, Biomedical engineering, Electrochemistry, Neuroscience

## Abstract

Recent work has shown the ability to record neural behaviour in pre-clinical studies from an endovascular location for over a year. Previous work on stimulating neural tissue from an endovascular location has also shown motor-evoked responses in sheep. However, endovascular stimulation requires high currents and can result in electrode degradation. This study aimed to modify an endovascular electrode to increase its charge injection capacity for efficacious neural stimulation. The platinum endovascular electrode was modified with platinum black and characterised by electrochemical and microscopic techniques. The stability of the electrode coating was assessed after a 7-day continuous stimulation paradigm. Modelling of the neural activating function was performed for central and peripheral neural anatomy with both electrode materials. Platinum black coatings had a substantially larger electroactive area than uncoated platinum. This resulted in increased electrode admittance, charge storage capacity and charge injection capacity while reducing the total impedance at 10 Hz and polarisation voltage. The coated electrode was comparatively more electrochemically stable than uncoated platinum following the 7-day continuous stimulation protocol. Modelling of the neural activating function indicated a substantial increase in the electrode-neuron distance which could be safely stimulated using platinum black coated electrodes. By comparison of electrochemical response with neural modelling, we have demonstrated the feasibility of safe stimulation of neural tissue using an endovascular neural interface, opening the possibility of a new, minimally invasive neural stimulation paradigm.

**Supplementary Information:**

The online version contains supplementary material available at 10.1038/s41598-025-93941-2.

## Introduction

Neural interfaces can be used to record neural activity to control prosthetic devices and provide important information on neural behaviour to advance our understanding of brain function. They can also be used to stimulate neurons, interrogate neural function and diagnose, monitor or treat multiple conditions including epilepsy, Parkinson disease and chronic pain.

Many people already receive benefits from cochlear implants, deep brain stimulation, spinal cord stimulators and vagus nerve stimulators using approved devices. These devices use relatively large electrodes that stimulate large regions of tissue. However, this can lead to off-target neural stimulation and negative side effects^1^. The large device size and need for open brain surgery when targeting the central nervous system can result in significant surgical trauma and limit the fidelity of neural recording and stimulation. Furthermore, encapsulation of electrodes with glial tissue can degrade device performance and increase power usage.

The endovascular neural interface allows electrodes to be placed in a blood vessel near the target tissue of interest via a catheter, eliminating the need for open brain surgery and direct physical contact with the delicate brain tissue or peripheral nerves^2^. Previous work on endovascular neural interfaces have reported on the safety of the surgical procedure^3^, the tolerance of blood vessels to the implanted device^4^ and similar signal quality to invasive arrays^5^. Being placed in a blood vessel, endovascular neural interfaces avoid fibrosis-related signal degeneration and enables long-term neural recording and stimulation^6^. The capacity has been demonstrated to record high-fidelity neural information over 20 weeks in animals comparable with commercially available implanted electrode arrays (Stentrode™, Synchron Inc., New York) and the use of this device in humans for over a year (brain recording clinical trial in progress NCT03834857)^7^. Endovascular brain signals recorded from an endovascular neural interface analyzed as functions of both time and electrode location were of comparable quality to commercial arrays^5^.

An endovascular neural interface has also been able to electrically stimulate the motor cortex of sheep^8,9^, inducing motor responses including involuntary twitching of the lip, neck, and jaw. However, the charge required for electrical stimulation to induce movement was significantly higher than the charge injection capacity (CIC) of the electrodes, resulting in electrode failure. Present generation endovascular arrays are made by thin film deposition methods, which typically have low CIC due to its smooth surface, reducing the effective surface area of the electrodes.

Previous approaches to increasing the CIC of neural interfaces have included increasing its capacitance via surface roughening (e.g. platinum black) and increasing its Faradaic charge capacity by coating with various materials (e.g. conducting polymers)^10^. The coating of electrodes with platinum black is usually performed by electrodeposition^11^ or sputter coating^12^. While electrodeposition generally creates more strongly bound and conductive coatings, sputter coating is generally easier to incorporate into manufacturing processes as it doesn’t require immersion of the device into solution and forming electrical contact with each electrode. Platinum black coatings have been utilised in a range of neural interfaces: they have been applied to microelectrode arrays for in vitro electrophysiological recording of myocytes, spinal cord neurons^13^, hippocampal neurons^14^ and cortical slices^15^. Platinum black substantially increased the CIC of a cochlear implant and was subsequently implanted into a feline model for chronic stimulation studies where the electrochemical benefits over uncoated platinum were maintained^16^. It has been used to coat microelectrodes for neural recordings in rat hippocampus^17^ and cerebral cortex^18^, and for electrical stimulation in the mid-brain of a fish^19^. It has also been applied to a cuff electrode for electrical stimulation of a rat sciatic nerve^20^. However, platinum black has not yet been applied to endovascular neural interfaces, and its capacity for providing clinically useful increases in CIC are not known.

This article investigates the feasibility of coating an endovascular neural interface with platinum black to increase its CIC. The electrochemical performances of uncoated and platinum black coated electrodes are assessed before and after a 7-day continuous stimulation protocol. The calculated CIC were then used to simulate the activation of central and peripheral nerves by electrical stimulation using these arrays.

## Experimental methods

### Electrochemistry

**Fig. 1:**
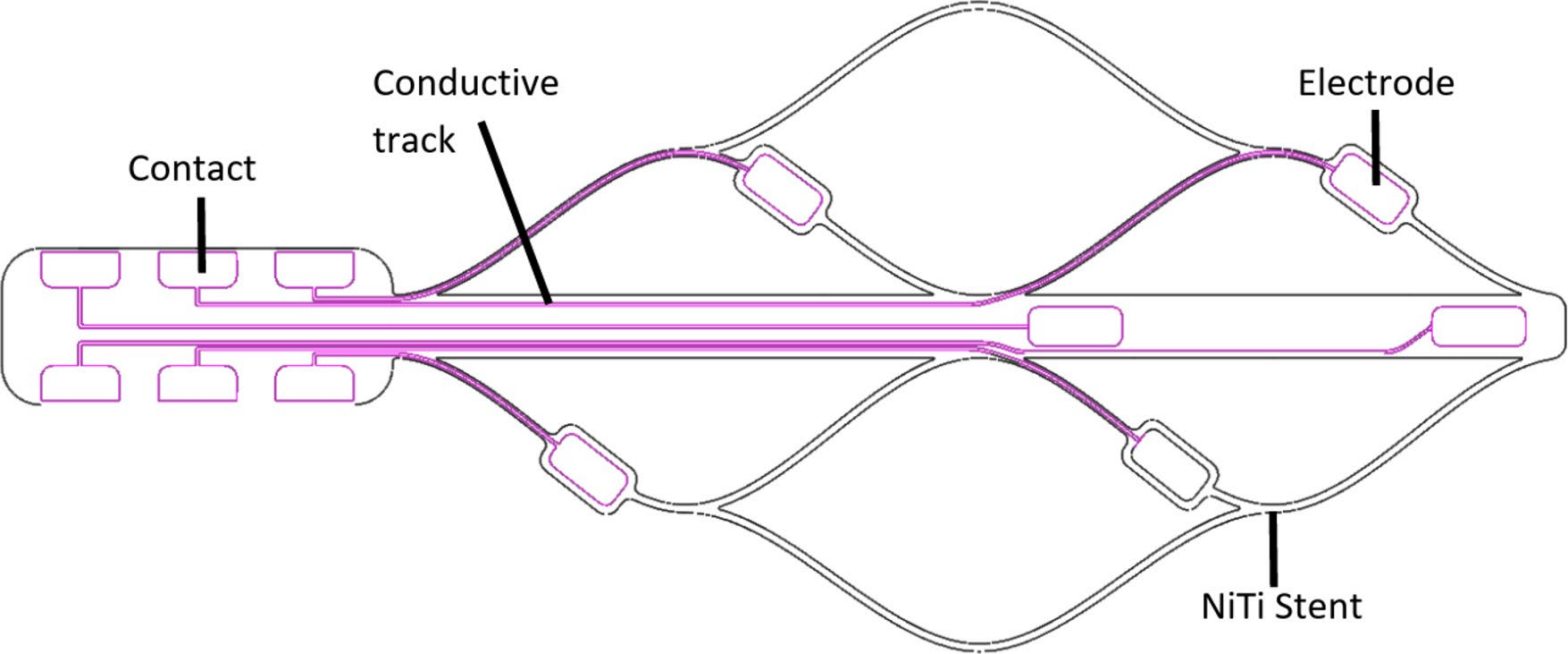
Platinum endovascular stent electrode design. The six electrodes placed on a NiTi substrate have platinum tracks to the connector tab.

Two electrode arrays (six electrodes per array) were manufactured by magnetron sputtering, UV photolithography and wet etching, followed by heat treatment to set the nitinol shape (Acquandas)^21,22^. The 300 nm thick platinum working electrodes had a 0.5 mm^2^ nominal area; the electrodes were connected via platinum conducting tracks sandwiched between two 250 nm thick layers of isolating zirconium oxide and attached to 50 μm thick NiTi stents (Fig. 1). One electrode array was subsequently sputter coated with 4 N platinum at high pressures to coat the smooth platinum with platinum black^12^ (this high purity platinum sputter avoids the use of Pb(NO_3_)_2_ in an electrodeposition process^11^). Wires were soldered to the electrode contacts and alligator clips used to connect to a potentiostat.

Electrochemistry was performed using a 1010E potentiostat (Gamry) in a three-electrode configuration. A Ag/AgCl (sat KCl) reference and platinum wire counter electrode far greater in size than the working electrode were used. Electrodes were not cleaned before use and were tested in non-degassed 0.9% saline (Baxter). The solution was not degassed as the electrode is placed in an endovascular location, where the oxygen tension is non-negligible^23^. Phosphate buffered saline was not used as phosphate adsorbs to platinum, altering the electrochemical response, and is a poor model of in vivo electrochemistry^24,25^.

Cyclic voltammetry was performed at 50 mV s^−1^ for two cycles from 0 → 0.8 → − 0.6 V. The anodic charge storage capacity (CSCa) was calculated from the anodic sweep of the first cycle (0 → 0.8 V), and the cathodic charge storage capacity (CSCc) on the cathodic sweep of the first cycle from the voltage on the voltammogram where it crossed 0 A, to − 0.6 V. The charge density was calculated from the CSC and nominal electrode area. Electrochemical impedance spectroscopy (EIS) was performed at 0 V with a 10 mV amplitude from 1 Hz to 200 kHz. Equivalent circuit fitting of the EIS was performed with Gamry Echem Analyst. Chronopotentiometric voltage transients (VT) were performed with a cathodic first, biphasic pulse of 250 µs phase length, with amplitude of 100, 150, 200 or 400 µA, no interphase gap, a 2 ms interpulse interval at 0 µA, with a sampling period of 10 µs; this equates to charge injection values of 5, 7.5, 10 and 20 µC cm^− 2^ per pulse, respectively. An average of 15 VTs was obtained from each stimulation amplitude on each electrode.

**Fig. 2:**
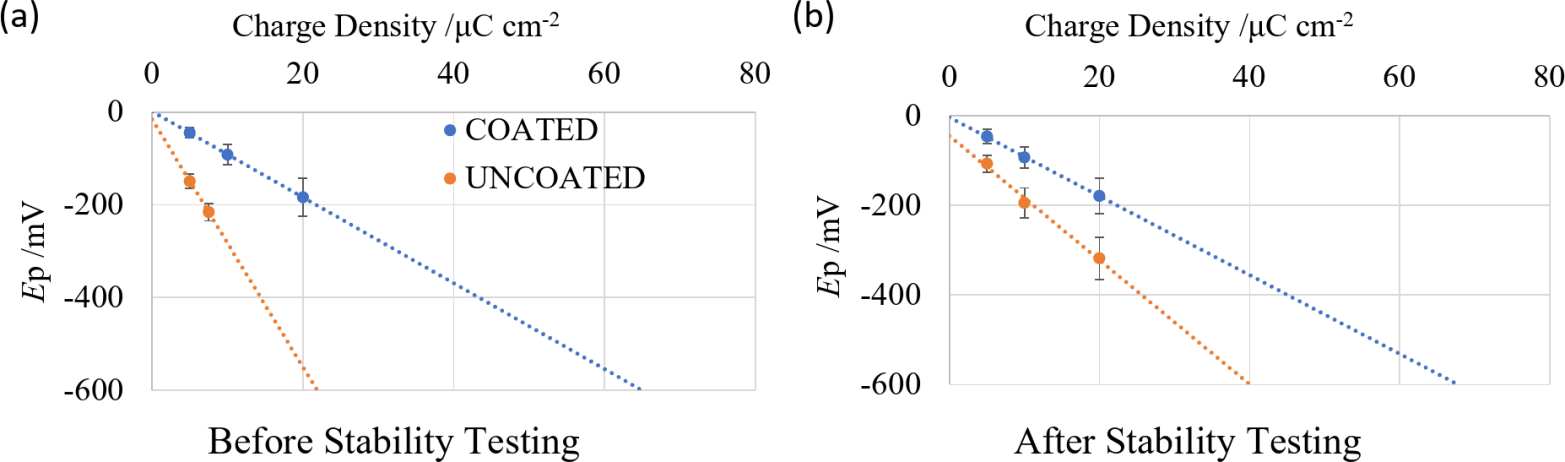
Determination of electrode charge injection capacity from charge density value at an *E*_p_ of − 600 mV (**a**) before and (**b**) after 7 days of stability testing.

The access voltage (*E*_a_), polarisation voltage (*E*_p_) and total voltage (*E*_t_= *E*_a_ + *E*_p_) were calculated with a slight modification to reference^26^. *E*_a_ was measured on the first cathodic pulse from the change in potential over four consecutive data points (30 µs), rather than two consecutive data points (10 µs); this was due to the Gamry potentiostat having a minimum sample time of 10 µs, while the purpose-built recorder used in reference^26^ had a minimum sample time of 5 µs. The maximum safe stimulation level was calculated with a slight modification to reference^26^. The *E*_p_ was determined for each VT current amplitude and plotted versus the applied charge injection value (Fig. 2). A linear trendline was then applied to determine what charge injection value resulted in an *E*_p_ of -600 mV (the CIC); in contrast, reference^26^ applied charge densities up to and above an *E*_p_ of -600 mV. While the extrapolation method slightly reduces the accuracy of the CIC measurement, it prevented damage to the electrodes from applying large currents. The maximum CIC was 21.9 µC cm^−2^ for the initial uncoated platinum electrodes, and 64.9 µC cm^−2^ for the initial platinum black coated electrodes. These values are calculated from the average of 15 VT on 6 electrodes; the CIC of an individual electrode differs slightly from the average and changes over time.

Following electrochemical characterisation, electrodes were subjected to a continuous stimulation paradigm for 7 days using a PlexStim Electrical Stimulator (Plexon) in non-degassed saline. The Plexon stimulator had a maximum output amplitude of 1 mA; therefore, the VT waveform used earlier was adjusted to a longer phase length at lower amplitude (same charge). The stimulator also required an interphase gap. A cathodic first, biphasic pulse, 300 µs phase width, 50 µs interphase gap, 500 Hz pulse frequency was applied. The pulse amplitude was adjusted to 0, 50 or 90% of the CIC. On uncoated platinum electrodes, the pulse amplitude at each level was 0, 183 or 330 µA, and for platinum black coated electrodes, was 0, 542 or 975 µA. Two electrodes were stimulated continuously at each pulse amplitude. The pulse amplitudes were calculated from the average CIC, and subsequently were slightly different percentages of an individual electrodes CIC. Electrodes were then retested by voltammetry, EIS and VT. Differences before and after continuous stimulation were assessed by paired two tailed *t* tests.

### Microscopy

Atomic force microscopy (AFM) images were obtained in contact mode on an MFP-3D (Asylum Research) with MLCT non-conductive silicon nitride cantilevers (Bruker). Helium ion microscopy (HIM) images were obtained on an Orion Nanofab (Zeiss) with an Everhart-Thornley secondary electron detector using an ion beam of 30 kV.

### Neural modelling

A Finite Element Method (FEM) tissue model was constructed using Sim4Life 7.2 (ZMT Zürich MedTech AG, Switzerland). The cortical model consisted of grey matter bulk tissue, a blood vessel with an outer radius of 2 mm and wall thickness of 0.5 mm, blood, an endovascular stimulating electrode and a large remote return electrode (Figure S1). The electrical properties of the materials were obtained from reference database IT’IS LF 4.0^6^. The axon splines in the model were represented using the Spatially Extended Nonlinear Node (SENN) neuron model with 5 μm fiber diameter. The axons and blood arteries were positioned perpendicular to each other, with the distance between them ranging from 0.5 mm to 2.5 mm in 0.1 mm increments relative to the stimulating electrode (Figure S2).

A FEM model for a peripheral neurovascular bundle was constructed separately with an arbitrary peripheral nerve of 1 mm diameter (Figure S3). The nerve was positioned immediately adjacent to the blood vessel with the endovascular electrode in an ideal position oriented towards it. The nerve model contained eight fascicles with diameters of 150–300 μm, which are common in fascicles of human somatic nerves^27–29^. The perineurium was modelled as a layer with a thickness of 3% of the fascicle diameter (4.5 μm and 9 μm)^30^. Endoneurium was modelled as all the material inside the fascicle, while epineurium was all the material between fascicles within the nerve bundle. The multicompartmental SENN model for myelinated fibers was used to simulate the dynamic neuronal responses of 200 axons evenly distributed in the fascicles^31^. All axon fibers were presumed to have a uniform diameter of 10 μm, a fiber diameter of high incidence in peripheral myelinated axons^28,32^. The model includes nodes of Ranvier with a nodal gap of 2.5 μm and 1 mm intermodal space (which is proportional to fibre diameter) with a perfectly insulating myelin sheath^31,33^. The conductivities of the inhomogeneous neural tissue are listed in Table S1. The spherical brain tissue environment was replaced by saline to simulate interstitial fluid.

To establish the boundary conditions, a constant potential of − 1 V was applied to the return electrode, while the stimulating electrode was set at 1 V. The electromagnetic low frequency (EM LF) ohmic quasi-static solver was used to solve the overall electromagnetic field within the tissue model. The resulting field was scaled according to the measured current flux passing through the electrode pairs, maintaining a constant total stimulation current of 1 mA.

Sim4Life’s built-in NEURON solver (Yale University, CT, USA) was directly linked with the computed electromagnetic field. A cathodic-first symmetric biphasic pulse of 250 µs phase length, with no inter-phase delay dynamically scaled the static potential field to match with the experimental stimulus waveform. The pulse amplitude was iteratively titrated to determine the minimum threshold for action potential generation. The activation threshold current for each axon was calculated by multiplying the titration factor by the stimulation current.

The activating function is conceptualized as the second derivative of the membrane potential along the axon, serving as an approximate model of the extracellular field’s influence on neurons. The activating function was approximated by the largest eigenvalue and corresponding eigenvector of the electric field gradient, derived by calculating the hessian matrix of second partial derivatives of the scalar electric potential field. Neural tissue activation was deemed to occur when the activating function surpassed the threshold of 26.66 V cm^−2^^34^.

## Results

### Imaging of uncoated and platinum black coated electrodes

**Fig. 3:**
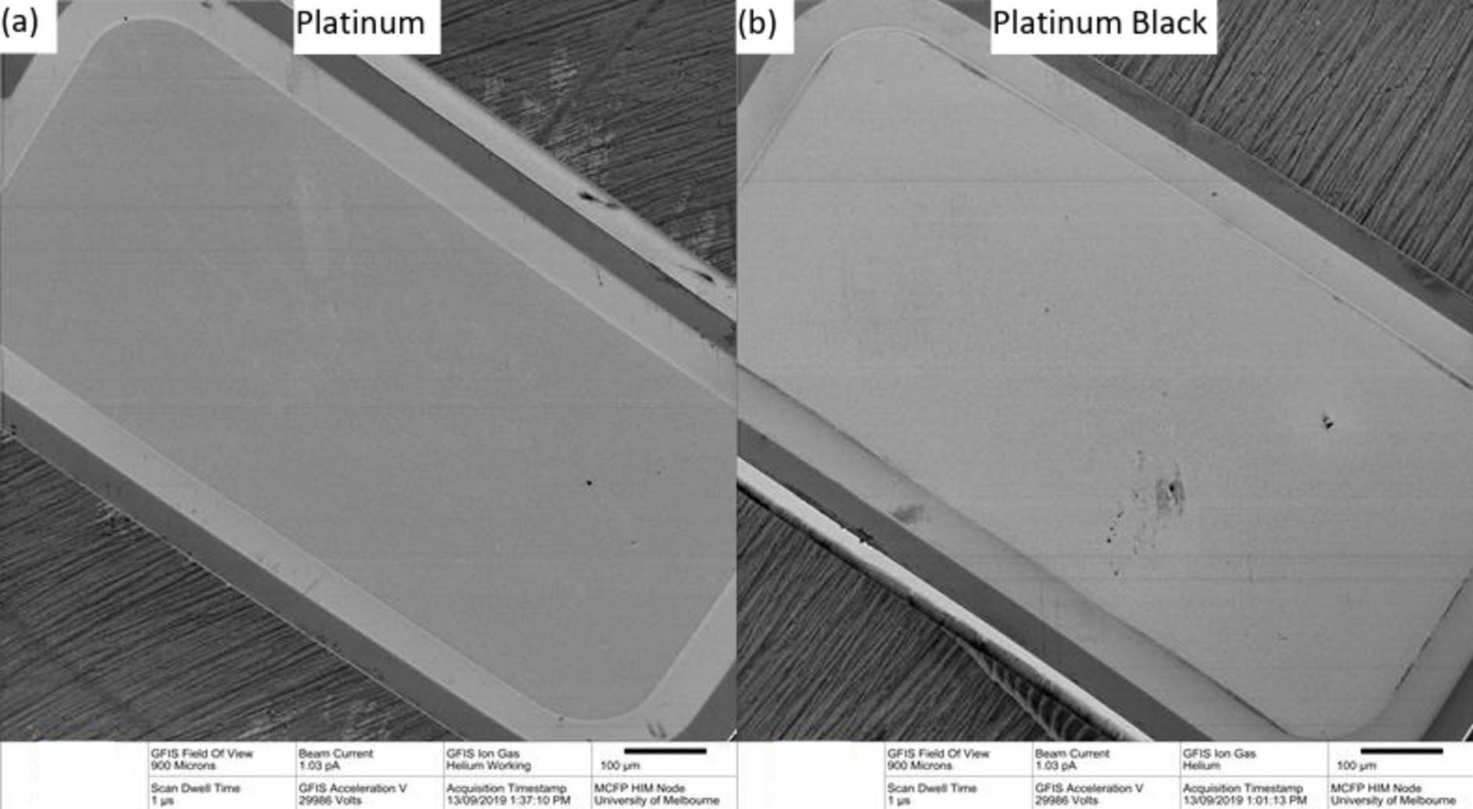

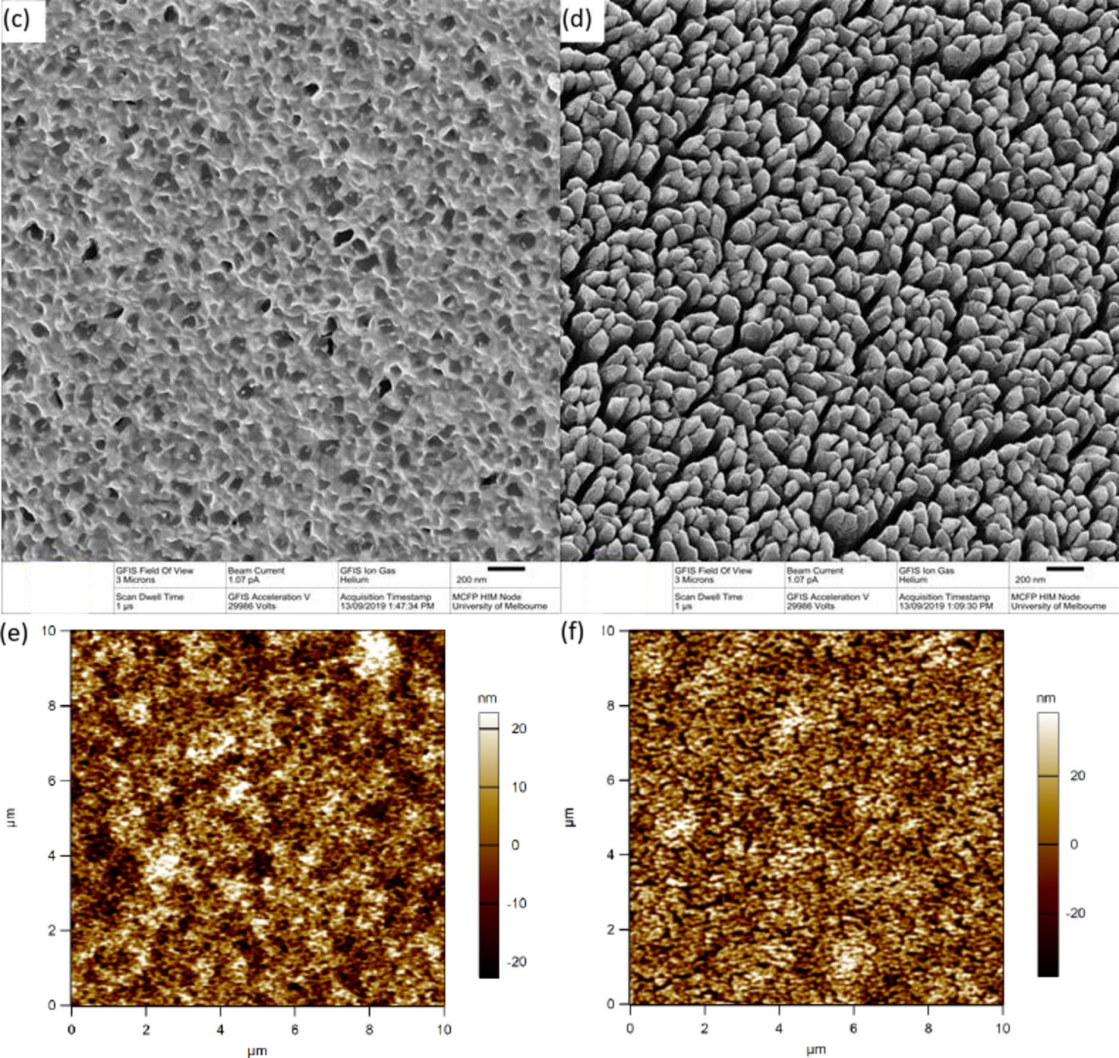
(**a**–**d**) Helium ion microscopy image and (**e**, **f**) atomic force microscopy of (**a**, **c**, **e**) uncoated and (**b**, **d**, **f**) coated platinum electrode.

The uncoated platinum electrodes were light grey pads attached to the flexible nitinol stent while platinum black coated electrodes were a darker colour. Under HIM (Fig. 3a–d), the uncoated electrodes showed microcrystalline surface structure (Fig. 3c) while the coated electrodes had a nodular appearance (Fig. 3d). AFM gave an RMS surface roughness of 11.79 ± 0.77 nm for uncoated electrodes and 18.60 ± 2.06 nm for platinum black coated electrodes (Fig. 3e,f).

### Initial electrochemical analysis of uncoated and platinum black coated electrode

**Fig. 4:**
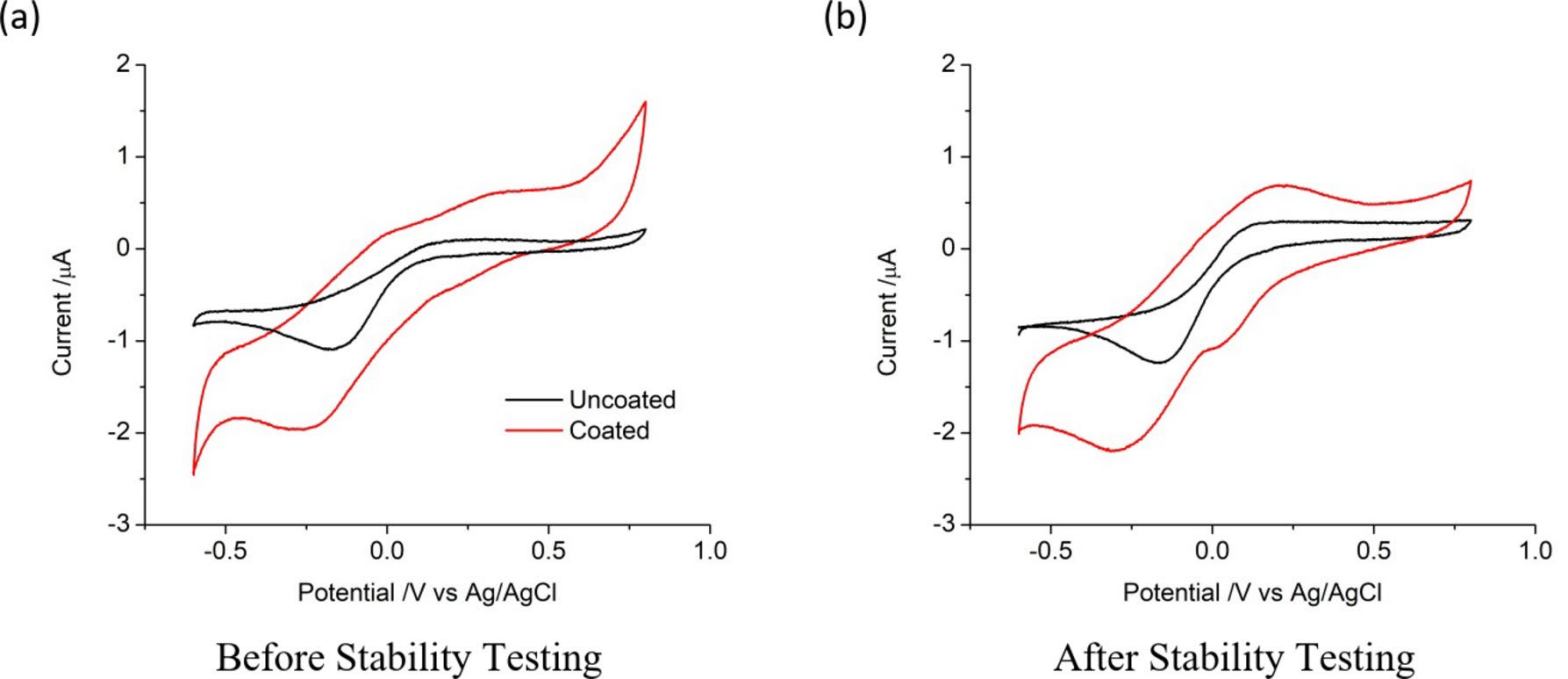
Representative second cycle of a cyclic voltammogram in saline of different platinum electrodes at 50 mV s^−1^. (**a**) before and (**b**) after 7 days of stability testing at 90% CIC.

Cyclic voltammetry was performed initially on each electrode in non-degassed saline. The response from the second voltammetric cycle at 50 mV s^−1^ of an uncoated platinum electrode displayed an irreversible reduction peak at – 171 mV (1.095 µA) with no oxidation processes (Fig. 4a). The irreversible reduction peak was mostly due to reduction of oxygen in the non-degassed solution^35^. Platinum black coated electrodes had a larger reduction current beginning at 410 mV and a broad peak at – 270 mV (1.963 µA); on the oxidation sweep, there were shoulders around 0 V and 340 mV. These responses are similar to previous reports of platinum and platinum black voltammetry^20,24^.

The CSC was measured for both electrode coatings; the value includes capacitive and Faradaic charge but contains different redox reactions on the oxidation and reduction sweeps^24,36^. Uncoated platinum had a CSCc of 17.7 and CSCa of 4.3 nC while platinum black had a CSCc of 24.5 and CSCa of 11.0 nC (Table 1). Using a nominal area of 0.5 mm^2^, the cathodic and anodic charge density were 3.5 and 0.9 mC cm^−2^ for uncoated platinum, and 4.9 and 2.2 mC cm^−2^ for platinum black. Coating the electrode with platinum black increased the CSCc 1.38 times and CSCa 2.56 times. This indicates platinum black more greatly enhanced the CSCa than CSCc within this potential range, enhancing the charge associated with oxidation reactions (e.g. platinum oxidation, chloride adsorption and water oxidation) more than the reduction reactions (e.g. oxygen reduction, hydride adsorption and water reduction). A comparison of platinum blacks’ CSC with previous literature was not possible, as these articles either do not report the CSC or calculate a single CSC value over both oxidation and reduction sweeps.

**Table 1:** Electrochemical measurements of uncoated and coated platinum before and after stimulation at varying current levels.

Electrode	Stimulation	Impedance 10 Hz/kOhm	Charge storage capacity/nC		Nominal charge density/mC cm^− 2^	
			Reduction	Oxidation	Reduction	Oxidation
Uncoated	Pre	36 (11)	17.7 (3.7)	4.3 (2.5)	3.5 (0.7)	0.9 (0.5)
Uncoated	0%	33 (3.3)	22.6 (8.9)	8.0 (0.6)	4.5 (1.8)	1.6 (0.1)
Uncoated	50%	21 (0.8)	32.0 (3.6)	10.8 (2.7)	6.4 (0.7)	2.2 (0.5)
Uncoated	90%	38 (7.6)	14.3 (3.8)	4.2 (0.5)	2.9 (0.8)	0.8 (0.1)
Coated	Pre	6.0 (1.9)	24.5 (6.8)	11.0 (1.9)	4.9 (1.4)	2.2 (0.4)
Coated	0%	8.7 (0.1)	29.2 (0.5)	13.9 (2.6)	5.8 (0.1)	2.8 (0.5)
Coated	50%	8.8 (1.7)	25.1 (1.8)	11.8 (2.3)	5.0 (0.4)	2.4 (0.5)
Coated	90%	6.0 (0.1)	29.1 (0.5)	9.2 (1.2)	5.8 (0.1)	1.8 (0.2)

Average (standard deviation) of 6 electrodes pre-stimulation and 2 electrodes post-stimulation.

**Fig. 5:**
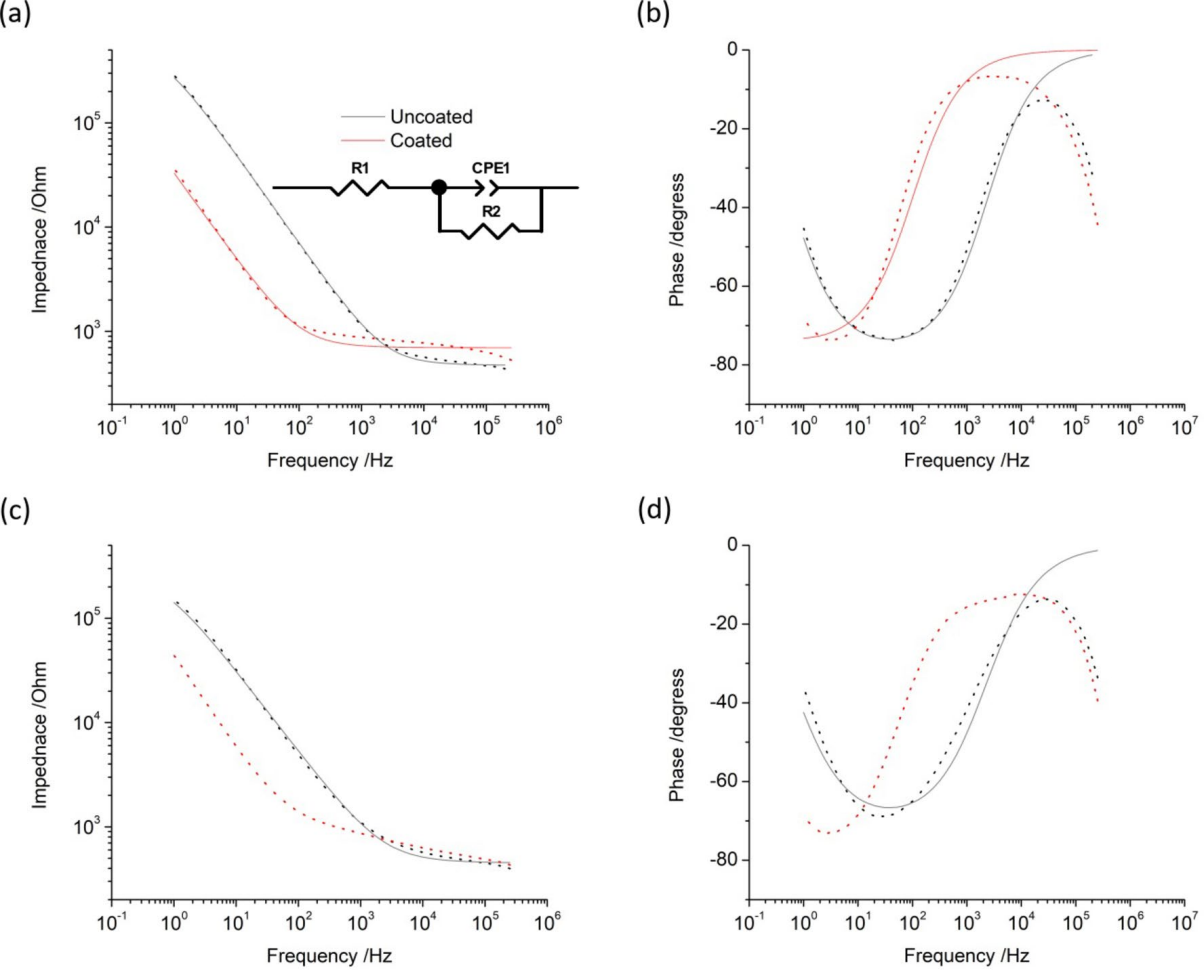
Representative electrochemical impedance in saline of different platinum electrodes at 0 V with an ac amplitude of 10 mV, (**a**, **b**) before and (**c**, **d**) after 7 days of stability testing at 90% CIC. Equivalent circuit used to model the impedance is shown. Dotted – data. Line – fitted response.

EIS was subsequently performed on each electrode at 0 V vs. Ag/AgCl in the same solution. This potential is used, rather than the open circuit potential (OCP), as 0 V vs. Ag/AgCl is defined and reproducible while OCP varies with conditions, resulting in an EIS response performed at different potentials for every experiment. At 0 V, charge transfer on the uncoated platinum was mainly associated with capacitance, while on the platinum black electrode, capacitive and Faradaic reactions could occur (Fig. 4a). The EIS displayed a single time constant for both uncoated platinum and platinum black (Fig. 5a,b) and was similar to previous reports^20,37,38^. At high frequencies, the impedance behaviour was dominated by the electrical properties of the solution, while the electrodes properties become more dominant in the low frequency response^39^. The shift in the platinum black EIS to lower frequencies and decrease in total impedance at low frequencies, compared to uncoated platinum, was consistent with an increase in effective electrode area. The total impedance at 10 Hz was highly correlated with electrode area and subsequently signal-to-noise ratio of neural recording (the commonly reported impedance at 1 kHz is less useful)^39,40^. Uncoated platinum had a total impedance at 10 Hz of 36 kOhm, while platinum black was 6 kOhm (Table 1).

An equivalent circuit was fit to the EIS response comprising resistors (R1 and R2) and a constant phase element (CPE1). R1 models the solution resistance, while the electrode/solution interface is modelled as a parallel constant phase element (CPE1) and polarisation resistance (R2). The constant phase element was used instead of a capacitor due to surface roughness or inhomogeneity in current distribution at the electrode surface. The quality of fit was greater for the uncoated platinum than platinum black. Values for the fitted resistance, admittance (*Q*_0_) and power (*n*) terms for the constant phase element are listed in Table 2. The solution resistance was within error for uncoated platinum and platinum black. The admittance of platinum black was 6.6 times larger than uncoated platinum, while the power term was within error. The charge transfer resistance of platinum black was 2.4 times larger than uncoated platinum and also had a substantially larger variation.

**Table 2:** Equivalent circuit fitting parameters of uncoated and coated platinum before, and uncoated platinum after stimulation at varying current levels.

Electrode	Stimulation	*R*_1_ /Ω	Q_0_ /10^− 7^ S s^1/2^	*n*	*R*_2_ /kΩ	χ^2^
Uncoated	Pre	598 (114)	9.7 (3.6)	0.82 (0.03)	349 (104)	0.029
Uncoated	0%	537 (25)	13.1 (2.7)	0.76 (0.03)	320 (19)	0.021
Uncoated	50%	414 (26)	23.3 (3.5)	0.73 (0.03)	223 (51)	0.027
Uncoated	90%	562 (157)	10.0 (2.8)	0.80 (0.01)	256 (34)	0.024
Coated	Pre	568 (135)	63.7 (3.7)	0.85 (0.01)	827 (537)	0.035

Average (standard deviation) of 6 electrodes pre-stimulation and 2 electrodes post-stimulation.

**Fig. 6:**
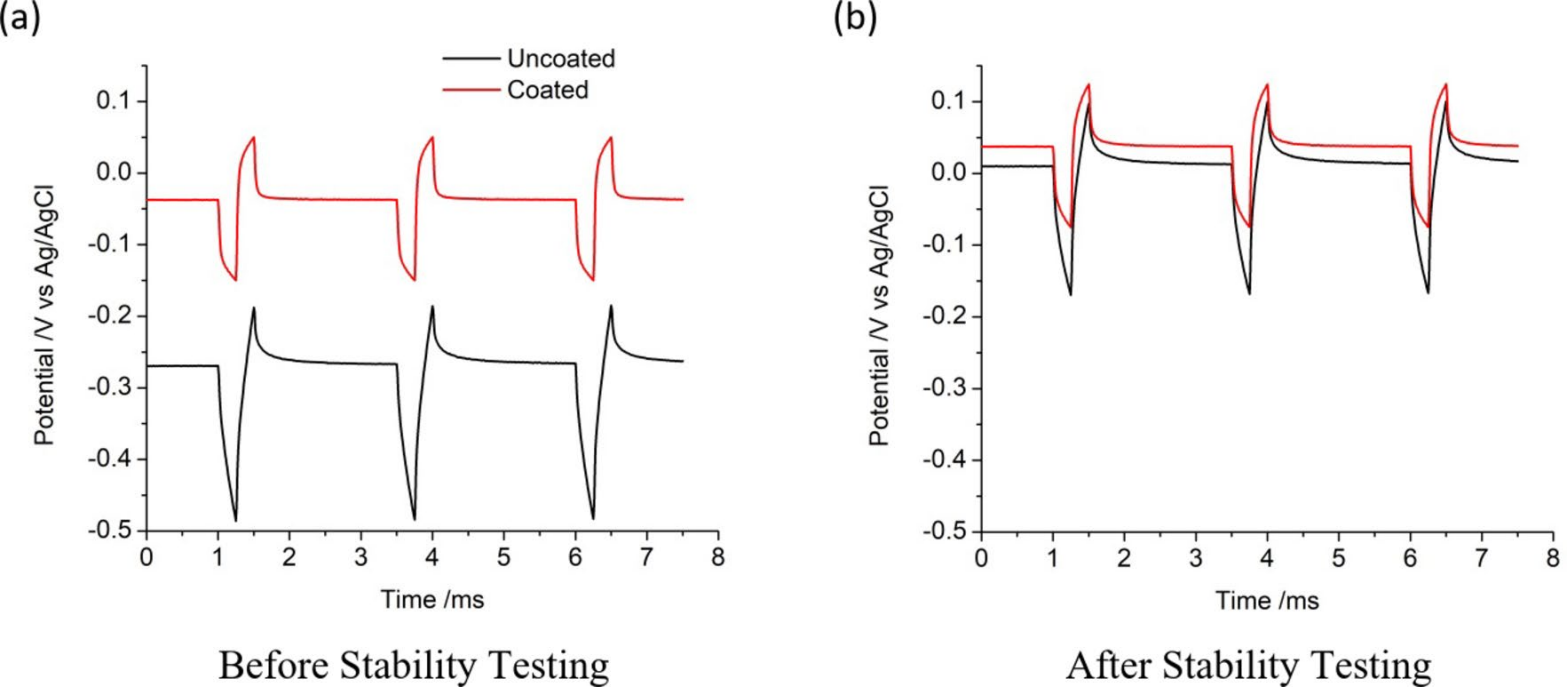
Representative multiple pulse chronopotentiometry in saline of different platinum electrodes, cathodic first 250 µs, 100 µA biphasic pulse with no interphase gap and 2 ms interpulse gap (**a**) before and (**b**) after 7 days of stability testing at 90% CIC.

Current pulses (VTs) were then applied to each electrode with a cathodic first biphasic pulse (Fig. 6a), as this is the waveform most commonly used clinically. The initial potential was the open circuit potential, which varied between electrodes and repetitions. Applying a cathodic first pulse, the electrode potential shifted closer to the water reduction limit, while repeated pulsing led to a ratcheting towards more positive potentials (due to unbalanced oxidation and reduction reactions, see reference^41^ for further discussions on this). The opposite trend occurred with an anodic first biphasic pulse. A single biphasic pulse was applied with a 2 ms interpulse interval, ensuring no potential ratcheting occurred. Three biphasic pulses were applied in an experiment, which was repeated five times for each electrode (15 repetitions in total), with *E*_t_, *E*_a_ and *E*_p_ calculated for each cathodic pulse. Over the 15 repetitions of a typical uncoated electrode with a 100 µA pulse amplitude, *E*_t_ increased from 194 to 197 mV, with *E*_a_ constant at 74 mV and *E*_p_ increasing from 120 to 123 mV. On a typical platinum black coated electrode, *E*_t_ increased from 144 to 151 mV, with *E*_a_ increasing slightly from 82 to 83 mV and *E*_p_ increasing from 62 to 68 mV. Increasing the pulse amplitude resulted in a linear increase in *E*_a_ and a slightly non-linear increase in *E*_t_ and *E*_p_. The average of all 15 repetitions on six electrodes with a 100 µA pulse amplitude is listed in Table 3. Compared to the uncoated platinum, platinum black coated electrodes had a slightly smaller *E*_a_ while *E*_t_ and *E*_p_ have decreased by ~ 100 mV. There was a greater difference in VT response between electrode coatings with increasing pulse amplitudes.

**Table 3:** Voltage transient measurements from the cathodic pulse of 100 ΜA biphasic pulse on uncoated and coated platinum before and after stimulation at varying current levels.

Electrode	Stimulation	Access voltage/mV	Polarisation voltage/mV	Total voltage/mV
Uncoated	Pre	79 (7)	147 (18)	225 (24)
Uncoated	0%	74 (4)	108 (2)	182 (2)
Uncoated	50%	63 (2)	80 (4)	143 (5)
Uncoated	90%	75 (9)	123 (11)	198 (20)
Coated	Pre	68 (17)	46 (11)	115 (27)
Coated	0%	60 (9)	43 (2)	103 (11)
Coated	50%	81 (7)	54 (2)	135 (6)
Coated	90%	52 (14)	41 (7)	93 (21)

Average (standard deviation) of 3 biphasic pulses on 6 electrodes pre-stimulation and 2 electrodes post-stimulation.

*E*_a_ is caused by uncompensated resistance (*iR*_u_), allowing application of Ohm’s law to calculate the electrochemical cell resistance. This gave 790 Ω and 680 Ω for uncoated and platinum black coated electrodes, respectively. These values were greater than the solution resistance values determined by EIS (Table 2). The expected *E*_a_ calculated from the EIS solution resistance would be 60 and 57 mV for uncoated and platinum black coated electrodes, respectively. Application of Ohm’s law to *E*_t_ and *E*_p_ is not valid, as these terms include Faradaic current^41^.

The CIC was determined by plotting *E*_p_ versus the pulse amplitude and extrapolating to the cathodic water reduction potential (Fig. 2a). With a cathodic first biphasic pulse, the water reduction limit occurred at lower pulse amplitudes than the water oxidation limit^41^. With an anodic first pulse, the water oxidation limit would occur at lower pulse amplitudes than the water reduction limit. Therefore, the CIC of a charge balanced, cathodic first biphasic pulse was defined by the water reduction potential. For uncoated platinum electrodes, this was 21.9 µC cm^−2^, and for platinum black coated electrodes, it was 64.9 µC cm^−2^. As described in the experimental section, these values were the average of 15 repeats and 6 electrodes, whereas the actual CIC (*E*_t_ and *E*_p_) of an individual electrode varied between experiments and electrodes.

Overall, the electrochemical data imply that platinum black coated electrodes should be able to safely inject a larger amount of current than uncoated electrodes.

### Analysis of uncoated and platinum black coated electrodes following 7 days stability testing

**Fig. 7:**
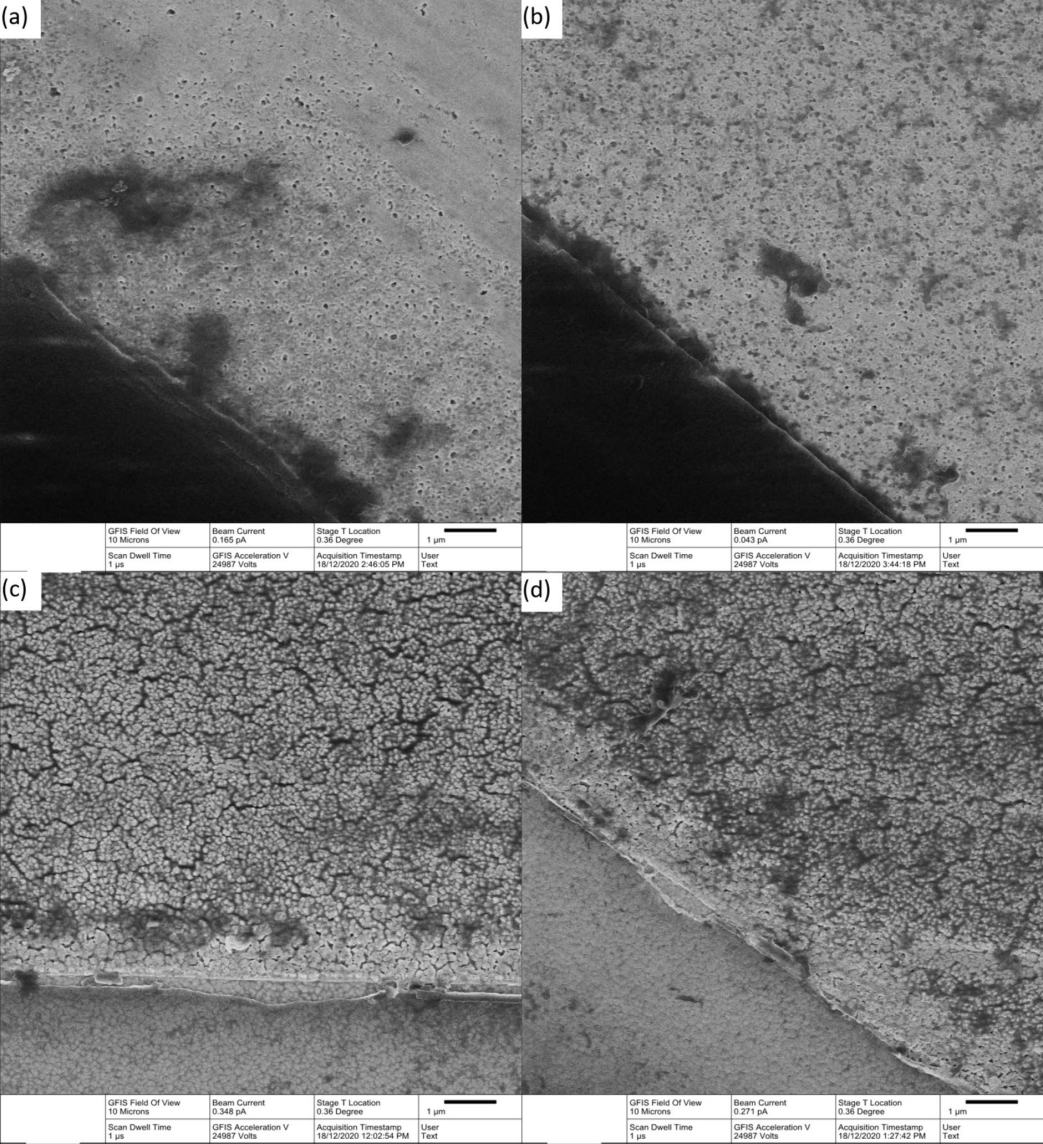
Helium ion microscopy image of (**a**, **b**) uncoated and (**c**, **d**) coated platinum electrode after 7 days of stability testing at (**a**, **c**) 0% and (**b**, **d**) 90% CIC.

Following continuous stimulation at 0%, 50% or 90% of the CIC for 7 days, electrodes were reanalysed by HIM and electrochemical techniques. The 7 day protocol provides sufficient information for assessing electrode stability during acute neural stimulation studies; however, a longer testing protocol will be required for assessing stability during chronic neural stimulation. HIM showed some change in electrode surface structure from their initial state, particularly at the electrode edges (Fig. 7). However, there was no substantial HIM difference between electrodes stimulated at 0% and 90% CIC. When current is passed through an electrode, the charge density is highest at the edges. This would suggest that the change in structure was caused by an electrochemical method, most likely during the slow scan rate cyclic voltammetry performed up to the water window, with less impact occurring during the stability testing applied at potentials below the water limit.

Cyclic voltammetry of the same uncoated electrode as described above was performed after stability testing at 90% CIC (Fig. 4b). The irreversible reduction peak shifted slightly to -163 mV and increased in magnitude to 1.242 µA with still no oxidation processes present. The same platinum black coated electrode as described above had a reduction shoulder around 23 mV and the reduction peak shifted to -303 mV and increased in magnitude to 2.200 µA. On the oxidation sweep, there was now a peak at 207 mV (0.686 µA).

The CSCc, CSCa and nominal charge densities of the uncoated electrodes increased from the initial values after stimulation at 0% and 50% CIC, while stimulation at 90% CIC was similar to the initial values (Table 1). In contrast, platinum black coated electrodes were consistent across the entire testing protocol.

EIS was performed on the same electrodes following stability testing and was very similar for the uncoated platinum, while the platinum black electrode had a slight change in response between 400 Hz and 25 kHz (Fig. 5c,d). The total impedance at 10 Hz was variable and mostly within error (Table 1). The same equivalent circuit could be fit to the uncoated platinum, but the change in shape of the platinum black electrode prevented fitting any reasonable equivalent circuit and subsequent comparison of fitted results. The solution resistance and power term of the uncoated platinum decreased with stimulation at 0% and 50% CIC but increased with stimulation at 90% CIC being similar to the initial electrode (Table 2). The admittance increased up to stimulation at 50% CIC then decreased with stimulation at 90% CIC, again similar to the initial electrode. The charge transfer resistance was generally within error across the entire testing protocol.

VTs were performed on the same electrodes following stability testing (Fig. 6b) with mean and SD of *E*_a_, *E*_p_ and *E*_t_ listed in Table 3. For the uncoated platinum, comparisons before and after stability testing showed significant differences in *E*_a_ at 0% (*t*(5) = 2.57; *p* = 7.0 × 10^−3^), 50% (*t*(5) = 2.57; *p* = 1.9 × 10^−4^) and 90% (*t*(5) = 2.57; *p* = 7.4 × 10^−6^) CIC, in *E*_p_ at 0% (*t*(5) = 2.57; *p* = 2.7 × 10^−4^), 50% (*t*(5) = 2.57; *p* = 4.3 × 10^−5^) and 90% (*t*(5) = 2.57; *p* = 7.2 × 10^−5^) CIC, and in *E*_t_ at 0% (*t*(5) = 2.57; *p* = 5.1 × 10^−4^), 50% (*t*(5) = 2.57; *p* = 6.1 × 10^− 5^) and 90% (*t*(5) = 2.57; *p* = 4.5 × 10^−5^) CIC. For platinum black coated electrodes, comparisons before and after stability testing showed significant differences in *E*_a_ at 50% (*t*(5) = 2.57; *p* = 1.8 × 10^−3^) and 90% (*t*(5) = 2.57; *p* = 7.1 × 10^−3^) CIC, in *E*_p_ at 50% (*t*(5) = 2.57; *p* = 3.0 × 10^−6^) CIC and in *E*_t_ at 50% (*t*(5) = 2.57; *p* = 5.3 × 10^−5^) and 90% (*t*(5) = 2.57; *p* = 3.7 × 10^−2^) CIC.The CIC was recalculated by plotting *E*_p_ versus biphasic pulse amplitude and extrapolating to the cathodic water reduction potential (Fig. 2b). Uncoated platinum electrodes had an increase in CIC to 40.1 µC cm^−2^, while platinum black coated electrodes were relatively constant at 67.8 µC cm^−2^. Figure 2b averages all six electrodes stimulated at different percentages of CIC amplitudes. Separation of the data into different stimulation amplitudes showed no difference in CIC value. This again suggests the main changes in electrochemical response are due to the slow scan rate cyclic voltammetry, and not the 7-day stability testing at different stimulation intensities.

### Neural modelling of uncoated and platinum black coated electrodes

**Fig. 8:**
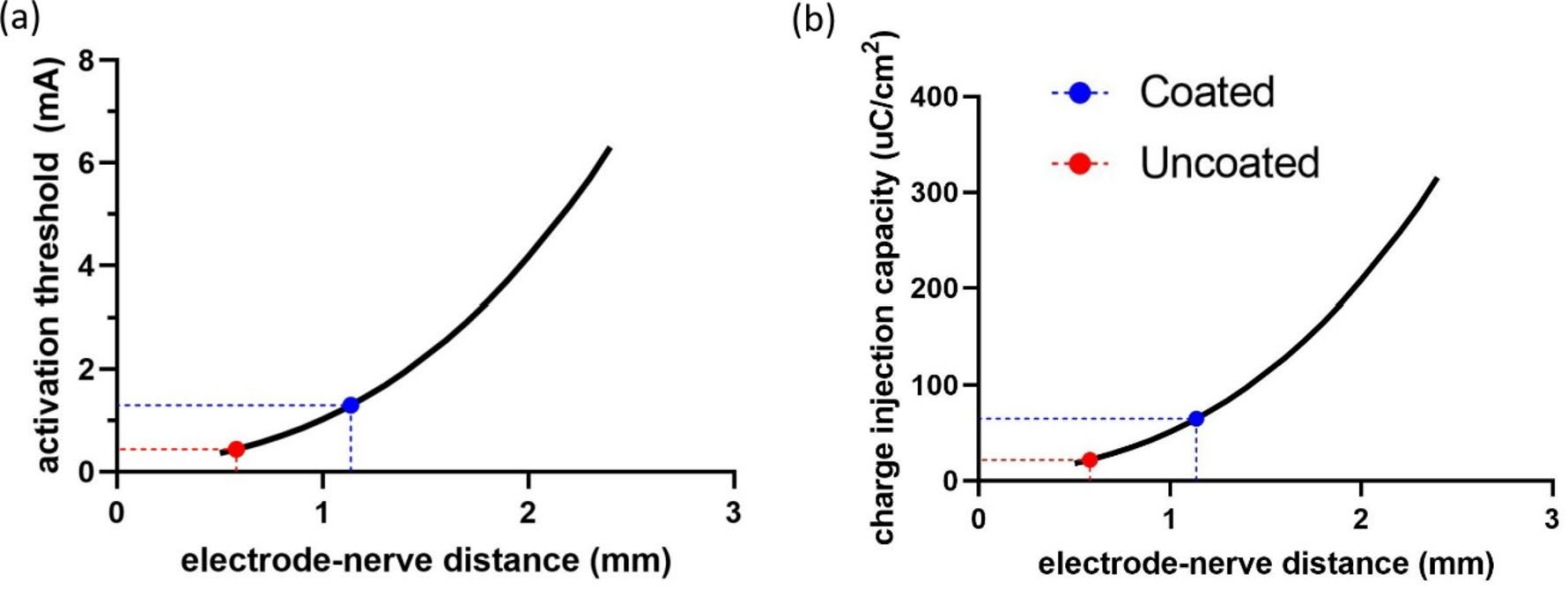
Neural modelling of cortical tissue with a parallel alignment of the blood vessel and neural fibre to determine the (**a**) activation threshold required to stimulate the nerve and (**b**) CIC required to safely stimulate the nerve with increasing electrode-nerve distance. The maximum safe stimulation distance that could be achieved with platinum or platinum black coated electrodes based on measured CIC is marked.

**Fig. 9:**
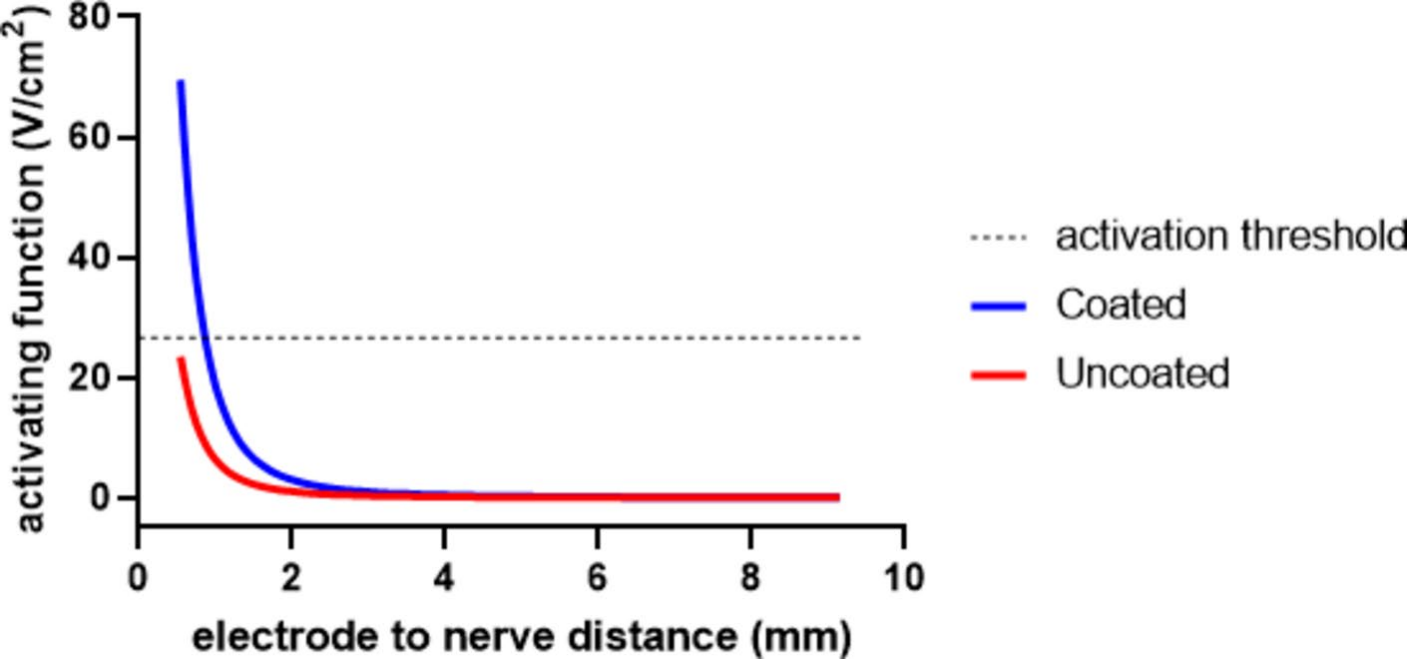
Neural modelling of cortical tissue with a parallel alignment of the blood vessel and neural fibre comparing the activating function versus electrode-nerve distance at the CIC for both uncoated and coated electrodes. The dashed line represents the neuronal activation threshold of 26.66 V cm^−2^.

Platinum black has been applied previously to a range of neural electrodes to enhance their CIC^13–20^, but has not been applied to endovascular electrodes where the electrode-neuron distance may be significantly greater. The ability to stimulate neural activity using uncoated and platinum black coated endovascular neural interfaces was assessed for cortical and peripheral neurons. For cortical neurons oriented perpendicularly to the stimulating electrode, the current required to activate the neurons increased with increasing electrode-neuron distance (Fig. 8a). The electrodes CIC needed to achieve neural activation also increased with increased electrode-neuron distance (Fig. 8b). Coating the electrode with platinum black increased the distance at which neurons can be safely stimulated from 0.58 mm to 1.14 mm. The same response was seen when the neurons were in a parallel orientation towards the electrode (data not shown). The activating function is a model of the potential across the neural membrane during electrical stimulation, which decreases with increasing electrode-neuron distance (Fig. 9). The model indicated the uncoated electrode would be unable to achieve a safe neural activation threshold from an endovascular location, while the platinum black coated electrode would be capable of stimulating neurons.

**Fig. 10:**
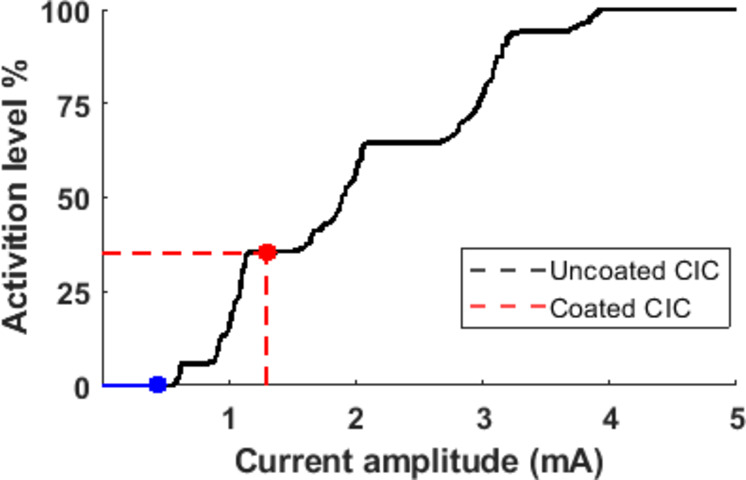
Neural modelling of a peripheral nerve bundle demonstrating axon activation level with increasing stimulating current amplitude. The uncoated electrode at CIC threshold is not able to recruit any axon, whereas the coated electrode yielded 35.3% activation of the total axon population.

**Fig. 11:**
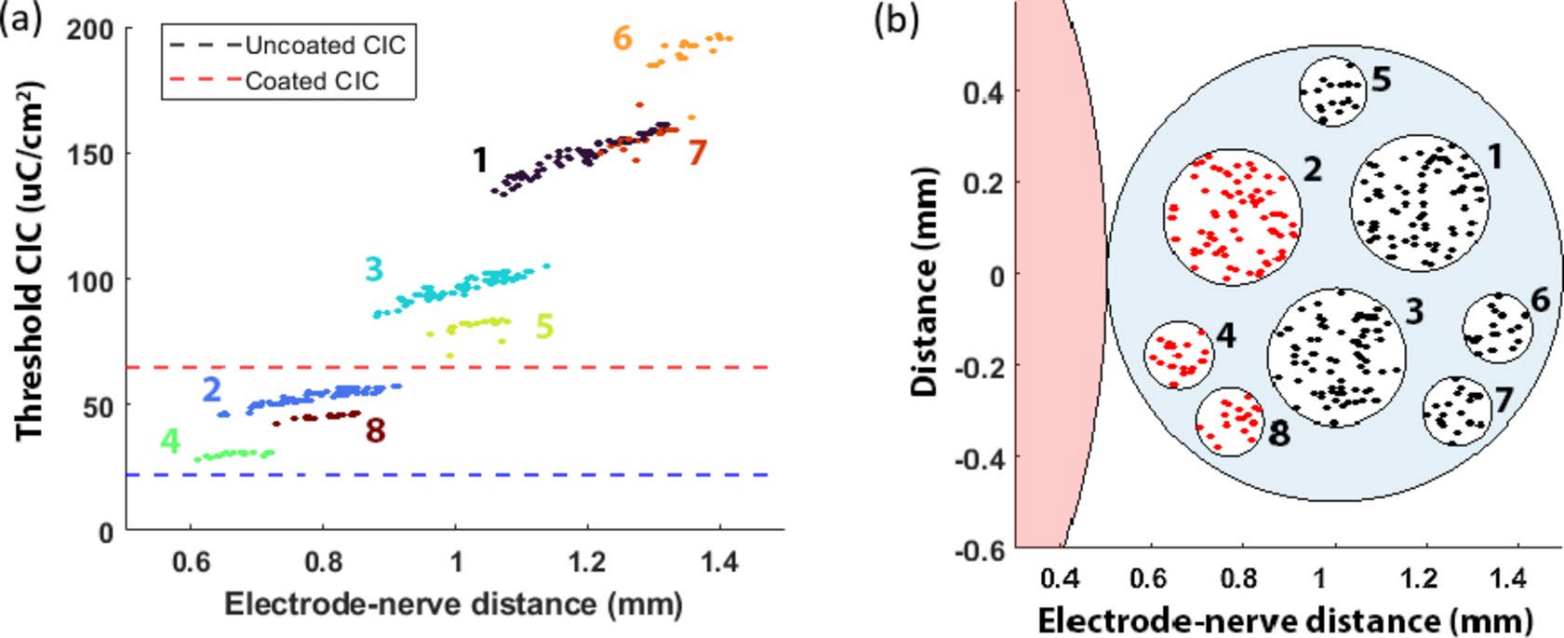
Neural modelling of a peripheral nerve bundle demonstrating the (**a**) threshold charge density of individual axon fibers versus electrode-nerve distance. Dashed lines indicate CIC values of the uncoated and coated electrodes. (**b**) Nerve cross-section showing activated axons (marked in red) at the CIC of a coated electrode. Indices represent individual fascicles.

For peripheral nerves, an arbitrary nerve bundle containing eight fascicles was modelled in a perpendicular orientation to the stimulating electrode. Increasing the stimulating current activated greater numbers of nerve fibres (Fig. 10). At the CIC, the uncoated electrode was unable to stimulate any nerves, while the platinum black coated electrode was able to activate 35.3% of the nerves. Plotting a cross section shows smaller fascicles with lower electrode-neuron distance are being activated (Fig. 11).

## Discussion

### Electrochemical performance of uncoated and platinum black coated electrodes

We have shown that it is possible to create an endovascular electrode array with a platinum black coated electrode surface. The high surface area coating significantly increases the electrodes admittance, CSC and CIC, while reducing the total impedance at 10 Hz and *E*_p_.

Following 7 days of electrical stimulation at varying current intensities, there were some general correlations in electrochemical parameters across each electrode coating, with total impedance at 10 Hz, solution resistance from EIS, and *E*_a_ following similar trends, while CSCc, CSCa, admittance and *E*_p_ were also highly correlated.

Overall, the CSCc, CSCa and admittance of platinum black were substantially larger than uncoated platinum, while *E*_p_ and subsequently *E*_t_ were smaller. There was a larger difference between electrode coatings from the fast measurements (short timescale) of admittance, *E*_p_ and *E*_t_ in comparison to slow measurements (long timescale) of CSCc and CSCa.

Timescale affects electrochemical response as surface roughness becomes smaller than the diffusion length of the ions in the surrounding solution. Subsequently, surface roughness becomes invisible at long timescales. For example, the diffusion length, (2*Dt*)^1/2^, for a sodium ion (diffusion coefficient = 1.33 × 10^−5^ cm^2^ s^−1^) during a 250 µs pulse would be ~ 0.8 μm. The time scale can be estimated during a voltammogram from the scan rate (*ʋ*) as 1/*ʋ*, giving a diffusion length of (2D/*ʋ*)^1/2^. Therefore, a sodium ion diffusion length during a 50 mV s^−1^ voltammogram would be ~ 23 μm. The scan rate would need to be over 40 V s^−1^ to create a diffusion length approaching 0.8 μm. However, higher voltammetric scan rates increase capacitance current and *iR*_u_, which can distort the voltammetric response, affecting any analysis such as CSC measurements and result in meaningless data. As a result, caution should be taken when using very high scan rate voltammetry for characterising neural electrodes.

There may be some change in surface structure and oxidation of the uncoated platinum when applying VTs up to 50% CIC, which subsequently increased its CIC. However, at 90% CIC, there may be some degradation of its electrochemical performance, leading to a response which was equivalent to the initial electrode state. Platinum black was generally electrochemically stable over the entire testing protocol, despite some visible changes via HIM. However, the inability to fit the platinum black EIS following 7 days of stimulation indicates the surface has become more heterogeneous. The change in EIS response highlights the limitations in measuring gross values such as CSC and CIC. The CSC and CIC measurements cannot explain or detect any changes to underlying charge transfer reaction mechanisms. For example, there may be a concurrent enhancement of surface (pseudo)capacitance and decrease in effective electrode area, which can cancel each other out in a CSC or CIC measurement.

The electrochemical data implies that the use of high surface area platinum black slightly reduces the resistance, but substantially reduces the amount of Faradaic charge passed during VTs (relatively more charge is being supplied by capacitance from the larger effective electrode area). Platinum black, therefore, has a larger CIC and is more stable. However, charge passed through the uncoated platinum electrode can increase its surface roughness or oxidation state, and subsequently its CIC, to some extent.

Similar electrochemical behaviour has been seen previously from application of platinum black to electrode surfaces, including increased surface roughness and CSC, and reduced impedance and *E*_p_^16,20^.

The electrochemical performance of the electrodes reported in this article was assessed in saline, which we have previously shown to be an appropriate model for in vivo performance^25^. However, protein adsorption will partially block the electrode surface, lowering its effective area, CSC and CIC^42^, Future studies will enhance our understanding of the impact biofouling plays on endovascular neural electrodes. Further limitations on the analysis of voltage transients for neural electrodes can been seen in our companion article^43^.

### Feasibility of neural stimulation using endovascular neural interfaces

An issue in the use of platinum black for neural interfaces is its potentially weak adhesion to the underlying substrate. This is of particular concern during electrode insertion, resulting in shear stress, and potential removal of the platinum black. However, in contrast to other bionic devices, the insertion of endovascular neural interfaces uses a catheter and vascular access sheath, eliminating any shear stress. A complete analysis of the mechanical properties of the platinum black before and after implantation will be published in a forthcoming article.

The endovascular neural interface is a promising approach for stimulating neural tissue without the need for highly invasive surgery. Blood vessels are in close proximity to all cell types in the body, so it may be theoretically possible to target any neuron from an endovascular location. This has been quantified in a mouse cortex, with the mean distance between neurons and the closest microvessel being 17.8 μm, with an average vessel diameter of 3.8 ± 0.3 μm^44^. For humans, the Fornix is a clinical stimulation target for the treatment of Alzheimer’s disease and the Subgenual cingulate white matter is the clinical stimulation target for treatment resistant depression. Access to the Fornix can be made via the internal cerebral vein (ICV), which is 1.7 mm from the Fornix and 1.2 mm from the pedunculopontine nucleus^45^. Subgenual cingulate white matter can be accessed through the A2 segment of the anterior communicating artery, which is 1.8 mm from the subcallosal cingulate gyrus and 0.8 mm from the pedunculopontine nucleus^45^. However, these blood vessels are small, with the ICV being 0.4–1.4 mm in diameter and the A2 segment of the anterior communicating artery being 1.9–2.2 mm in diameter^46^. This would require development of smaller endovascular neural interfaces than presented in this work and used in the Stentrode.

The neural models used in this work were of an arbitrary anatomy, suggesting that endovascular neural interface electrodes would need to be within a couple of mm of their target neurons. However, these models contain a number of assumptions and limitations. In reality, patient-specific neural anatomy will vary; for instance, they may have different fascicle diameters, numbers, arrangement, layer thicknesses, cortical structure and tissue conductivities. The model lacks any fibrous tissue or microhaemorrhaging, which may result from implantation. The model also does not take into account trauma or other disorders that may impact on anatomy or tissue function, or more complex biological mechanisms such as ephaptic coupling. It has also been found that endovascular neural interfaces are incorporated into the blood vessel wall, which would reduce the electrode-neuron distance. As a result, the exact activation threshold and safe stimulation distances within a patient (or animal model) will vary from the model predictions. For example, increased tissue conductivity would increase the volume of activated tissue. Differences in these factors would result in a variable degree of neural stimulation and impact on the clinical benefit of endovascular neural stimulation. Nevertheless, the model supports the conclusion that platinum black is required to achieve safe stimulation of neurons from an endovascular location. It may also be possible to use uncoated platinum electrodes above its safe CIC for acute research studies of neural stimulation. Overall, the ability of endovascular neural interfaces to be used for stimulating neural tissue will depend on the electrode size and CIC, blood vessel diameter and electrode-neuron distance. The blood vessel diameter and wall thickness, distance from the target tissue and the charge capacity of the electrodes may have to be assessed on a case-by-case basis.

Further work is still required for the development of a stimulating endovascular neural interface prior to its clinical application. The present study must be validated through acute animal studies. The 7 day stability protocol must be extended to assess the chronic stability of a stimulating electrode prior to chronic animal studies. These studies must evaluate the animals electrophysiological and behavioural response, as well as the biocompatibility and biostability of the electrode and tissue during endovascular stimulation. Further modifications of electrode design (size, coating material etc.) must then be undertaken as required. A review must be undertaken to determine the most suitable disease and anatomical target for first-in-human studies. There must also be a review of the ethical and regulatory issues associated with the development of this new medical device^47^.

## Conclusions

We have demonstrated the feasibility of coating an endovascular neural interface with platinum black, substantially increasing its effective area. The platinum black coating increased the electrodes admittance, charge storage capacity and charge injection capacity, while reducing its total impedance at 10 Hz and polarisation voltage. Neural modelling indicates platinum black substantially increases the electrode-neuron distance which could be safely stimulated in central and peripheral neural tissue.

## Electronic supplementary material

Below is the link to the electronic supplementary material.

Supplementary Material 1

## Data Availability

All data generated or analysed during this study are included in this published article.
