# Supplementary material for: Endovascular neural stimulation with platinum and platinum black modified electrodes
Source: Sci Rep. 2025 Mar 20;15:9676. doi: 10.1038/s41598-025-93941-2 (PMC11926064; doi:10.1038/s41598-025-93941-2)
Supplement: Supplementary file 1 — Supplementary Material 1 [file 41598_2025_93941_MOESM1_ESM.docx]

Supplementary Information

Endovascular neural stimulation with platinum and platinum black modified electrodes

Alexander R. Harris^1*^, Marko Ruslim^1^, Huakun Xin^1^, Zhiyi Shen^1^, JingYang Liu^1^, Tom Spencer^1^, David Garrett^2^, David B. Grayden^1,3^, Sam E. John^1*^

^1^ Department of Biomedical Engineering, University of Melbourne, Melbourne, 3010, Australia

^2^ School of Engineering, RMIT University, Melbourne, VIC 3001, Australia

^3^ Graeme Clark Institute, University of Melbourne, Melbourne, 3010, Australia

*Email: alexrharris@gmail.com sam.john@unimelb.edu.au


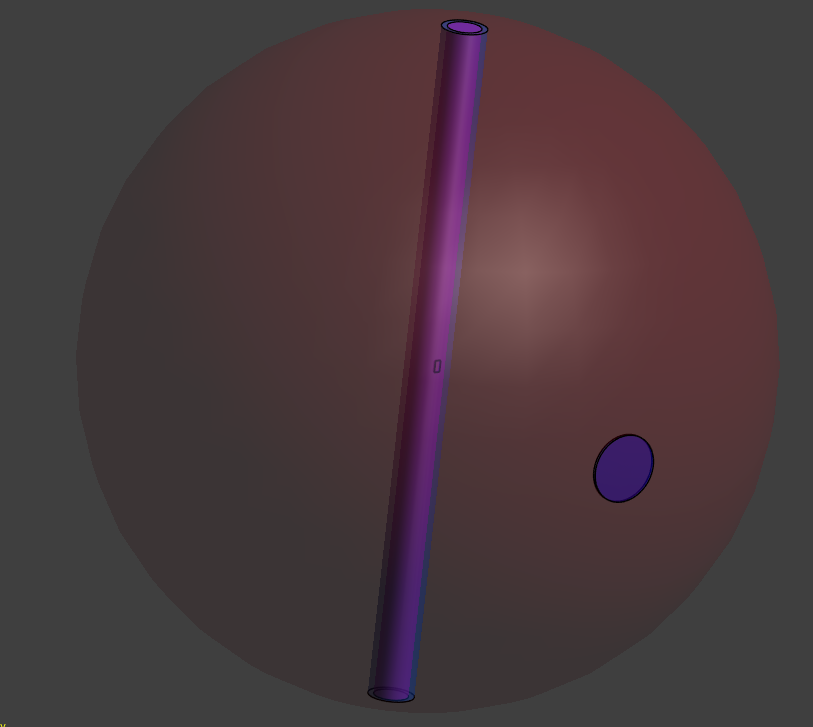


**Figure S1**: The FEM model consists of a brain tissue sphere, blood vessel, return electrode, and stimulating electrode.


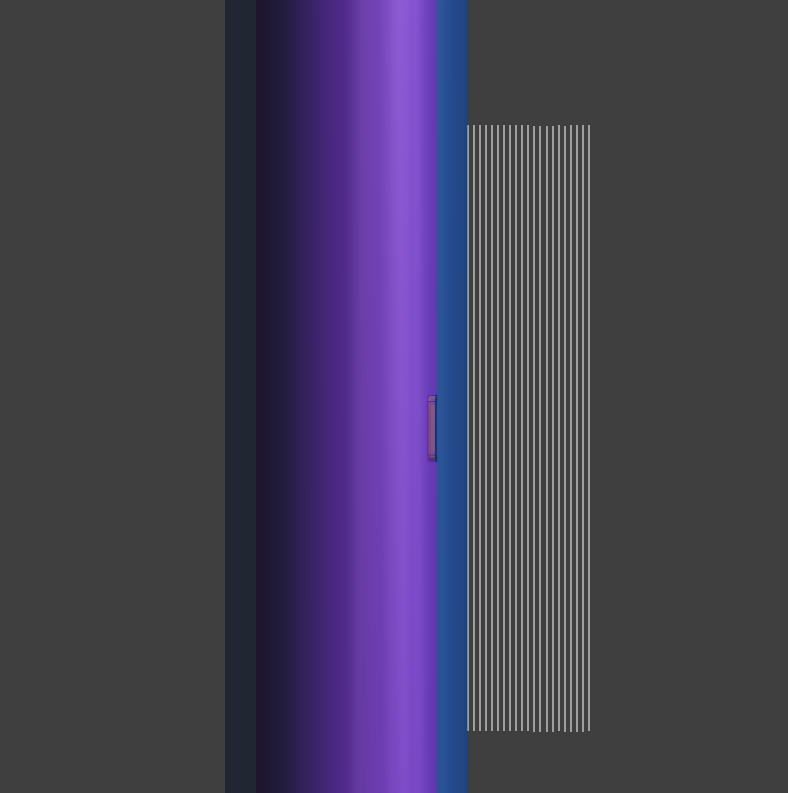


**Figure S2**: Orientation of SENN neurons in relation to blood vessels, demonstrating perpendicular alignment.


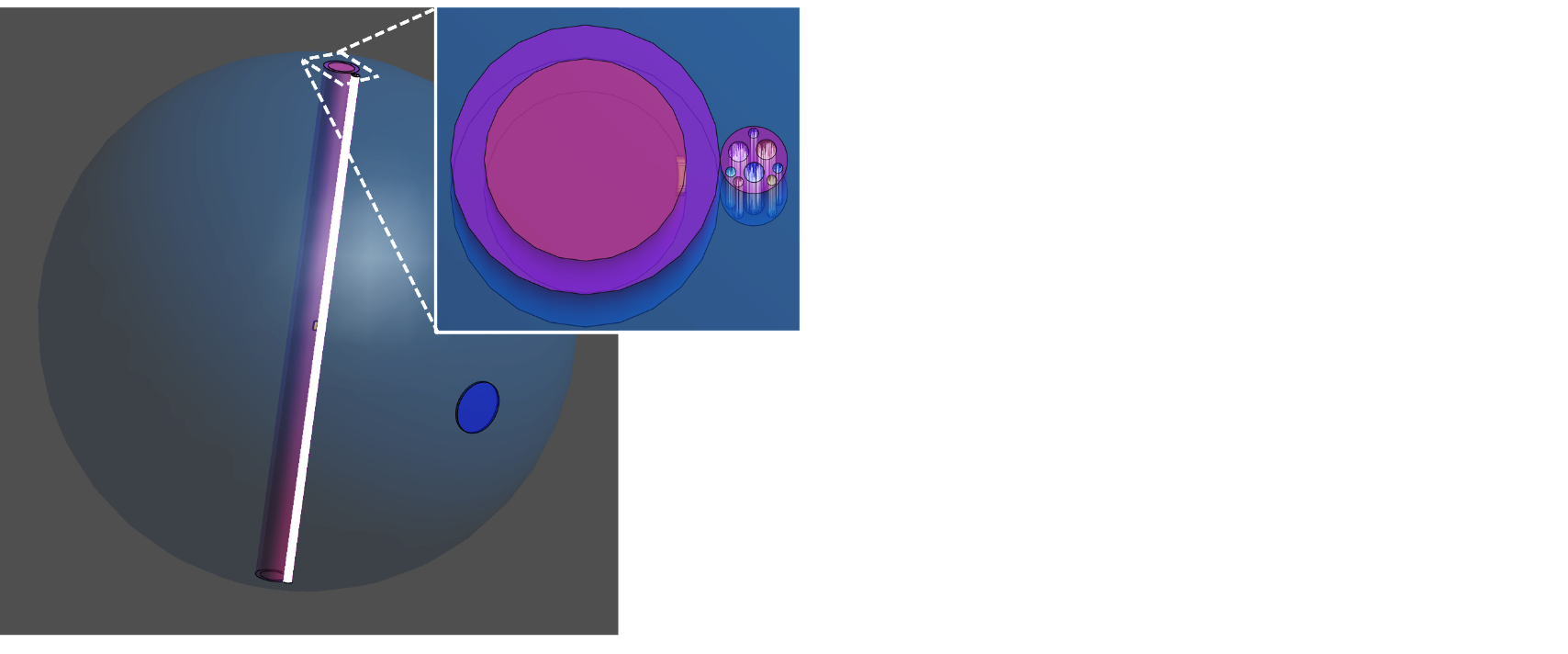


**A**

**B**

**Figure S3**: (a) FEM model of a peripheral nerve adjacent to a blood vessel, embedded in a spherical saline boundary. (b) Close-up of the highlighted region in (a). showing a cross-section of the neurovascular bundle, top view. Electrode position is indicated by yellow arrows.

**Table S1**: Electrical Properties of Materials

| Material | Electric Conductivity (S/m) | Relative Permittivity | Reference |
| --- | --- | --- | --- |
| Insulator | 0 | 1 |  |
| Blood | 0.659 | 5258.61 |  |
| Blood Vessel Wall | 0.232 | 149048 |  |
| Brain (Grey Matter) | 0.239 | 164063 |  |
| Saline | 2.0 | 1 |  |
| Epineurium | 0.083 | 1 | [1] |
| Perineurium | 0.00088 | 1 | [2] |
| Endoneurium (transverse) | 0.088 | 1 | [3] |
| Endoneurium (longitudinal) | 0.57 | 1 | [3] |

**References**

[1] Schiefer M A, Triolo R J and Tyler D J 2008 A Model of Selective Activation of the Femoral Nerve With a Flat Interface Nerve Electrode for a Lower Extremity Neuroprosthesis *IEEE Trans. Neural Syst. Rehabil. Eng.* **16** 195–204

[2] Raspopovic S, Petrini F M, Zelechowski M and Valle G 2017 Framework for the Development of Neuroprostheses: From Basic Understanding by Sciatic and Median Nerves Models to Bionic Legs and Hands *Proc. IEEE* **105** 34–49

[3] Eiber C D, Payne S C, Biscola N P, Havton L A, Keast J R, Osborne P B and Fallon J B 2021 Computational modelling of nerve stimulation and recording with peripheral visceral neural interfaces *J. Neural Eng.* **18** 66020
